# Does consanguinity increase the risk of bronchial asthma in children?

**DOI:** 10.4103/1817-1737.39634

**Published:** 2008

**Authors:** Mohammad I. El Mouzan, Abdullah A. Al Salloum, Abdulah S. Al Herbish, Ahmad A. Al Omar, Mansour M. Qurachi

**Affiliations:** *Department of Pediatrics, King Saud University, Riyadh, Saudi Arabia*; 1 *Department of Pediatrics, The Children's Hospital Medical Complex, Riyadh, Saudi Arabia*; 2 *Department of Pediatrics, Al yammama Hospital, Riyadh, Saudi Arabia*

**Keywords:** Bronchial asthma, consanguinity, Saudi Arabia

## Abstract

There is a high prevalence of consanguinity and bronchial asthma in Saudi Arabia. The objective of this study is to explore the effect of parental consanguinity on the occurrence of bronchial asthma in children. The study sample was determined by multistage random probability sampling of Saudi households. The families with at least one child with asthma were matched with an equal number of families randomly selected from a list of families with healthy children, the latter families being designated as controls. There were 103 families with children having physician-diagnosed bronchial asthma, matched with an equal number of families with no children with asthma. This resulted in 140 children with bronchial asthma and 295 children from controls. The age and gender distribution of the children with bronchial asthma and children from controls were similar. There were 54/103 (52.4%) and 61/103 (59.2%) cases of positive parental consanguinity in asthmatic children and children from controls respectively (*P* = 0.40). Analysis of consanguinity status of the parents of children with asthma and parents among controls indicates that 71/140 (51%) of the children with asthma and 163/295 (55.3%) of the children from controls had positive parental overall consanguinity (*P* = 0.43). The results of this study suggest that parental consanguinity does not increase the risk of bronchial asthma in children.

It is generally believed that bronchial asthma is caused by the interaction between genetic susceptibility and environmental exposure.[1] Recent research on the genetics of asthma indicated that there was an association between maternal asthma and increased total IgE levels. Further genetic studies have identified genes associated with asthma, and some other studies provided evidence of a major susceptibility locus located on chromosome 2p.[2,3] In Saudi Arabia, there is a high prevalence of both - bronchial asthma, reported to be 23% in 1995;[4] and consanguinity, reported to be between 54 and 56%.[5,6] Knowledge of the relationships between these two common conditions would be important for genetic counseling in relation to consanguinity. Therefore, our objective is to explore the impact of consanguineous marriage on the occurrence of bronchial asthma in children.

## Materials and Methods

The study sample was determined by multistage random probability sampling of Saudi households from representative regions of the country, used for the assessment of physical growth.[7] A questionnaire was designed for this purpose and administered to mothers during a household visit. Consent of the heads of households to participate in the study was obtained by the field teams. The mother in each household in the sample was asked about the relationship to her husband and to choose one of the three answers: first-degree cousin (including all four types), more distant relationship or no relationship. History and physical examination of all children and adolescents were performed by primary-care physicians, and physician-diagnosed cases of bronchial asthma were recorded. Subsequently, at the time of analysis, the families with at least one child with asthma (cases) were matched with an equal number of families (controls) randomly selected from a list of families with healthy children from the same regions. The data were analyzed using SPSS software to determine frequencies, and chi-square test was used to compare the proportions of cases and controls in relation to the risks of consanguinity. Separate analysis was performed to explore the effect of overall parental consanguinity and of the closest relationships, viz., first-degree cousin relationships, on the occurrence of asthma in children. A statistically significant difference was assumed when the *P* value was less than 0.05.

## Results

During the survey conducted over 2 years (2004-2005), 103 families with 140 children having physician-diagnosed bronchial asthma were identified and matched with an equal number of families of 295 healthy children with no history of bronchial asthma. There were 81/140 (57.9%) and 137/295 (46.4%) male children among the cases and controls respectively (*P* = 0.034). The age breakdown of the children with bronchial asthma and those from controls is shown in Table 1, indicating a similar distribution. The overall parental consanguinity status (all types of consanguinities) of the families with at least one child with bronchial asthma and control families is shown in Table 2, indicating that there were 54/103 (52.4%) and 61/103 (59.2%) cases of positive parental consanguinity in asthmatic and control children respectively (*P* = 0.40). Among the 54 consanguineous families of asthmatic children and 61 controls, 37 and 44 were first-degree cousins respectively. Analysis indicates that there were 37/86 (43.0%) and 44/86 (51.2%) cases of positive parental first-degree cousin consanguinity in asthmatic children and in children from controls respectively (*P* = 0.36). Table 3 presents a different way of data analysis - by looking at the parental consanguinity status of the children with bronchial asthma and of children from controls. There were 140 children with asthma matching with 295 healthy children of control families. Seventy-one out of 140 (51%) of the children with bronchial asthma and 163/295 (55.3%) of the control children had positive parental overall consanguinity (*P* = 0.43). Further analysis was performed to look at the relationship of parental first-degree cousin consanguinity to bronchial asthma in children. Table 4 indicates that 48 of the 71 asthmatic children and 117 of the 160 children from controls were offspring of first-degree cousin parents. Analysis indicates that parents of children with asthma did not have higher rate of first-degree cousin consanguinity than did parents among controls (*P* = 0.34).

**Table 1:** Bronchial asthma in children and overall parental consanguinity*

Age groups (Years)	Children with asthma No (%)	Controls No (%)
≤2	23 (16.4)	39 (13.2)
3–6	27 (19.3)	61 (20.7)
7–12	42 (30.0)	87 (29.5)
13–18	48 (34.3)	108 (36.6)
Total	140 (100)	295 (100)

* *P* = 0.40; odds ratio = 0.76; 95% confidence limit: 0.42–1.37

**Table 2:** Age distribution of children with asthma and those from controls

Consanguinity status of the parents	Health status of the families		Total
		
	Families of asthmatic children	Control families	
Consanguineous	54 (52.4%)	61 (59.2%)	115
Non consanguineous	49 (47.6%)	42 (40.8%)	91
Total	103	103	206

**Table 3:** Parental overall consanguinity and bronchial asthma in children*

Parental consanguinity	Health status of the children		Total
		
	Bronchial asthma	Healthy controls	
Positive	71 (51.0)	163 (55.3)	234
Negative	69 (49.0)	132 (44.7)	201
Total	140 (100)	295 (100)	435

* *P* = 0.43; odds ratio = 0.83; 95% confidence limit: 0.55-1.27; Figures in parentheses are in percentage

**Table 4:** Parental first-degree cousin relationship and bronchial asthma in children*

Parents consanguinity	Status of the children		Total
		
	Bronchial asthma	Healthy controls	
First degree cousins	48 (41)	117 (47)	165
Non-consanguinous	69 (59)	132 (53)	201
Total	117 (100)	249 (100z)	366

* *P* = 0.34; odds ratio = 0.78; 95% confidence limit: 0.49 and 1.25; Figures in parentheses are in percentage

## Discussion

In countries with high rates of parental consanguinity, knowledge of the risks to children is essential for appropriate counseling. Theoretically, consanguineous marriages should carry a high risk for the development of diseases that have a genetic basis, either completely or partially.[8,9] As a result, consanguinity is commonly blamed as one of the causes of genetically related conditions without proper evaluation. Such assumptions may adversely affect the application of genetic counseling and orient it against consanguinity.

Bronchial asthma is a good example of multifactorial diseases, which is common worldwide including countries with high rates of consanguinity. In this study, we have attempted to evaluate the role of parental consanguinity in the development of bronchial asthma in children. It was found that there was no statistically significant risk from parental consanguinity for the development of asthma in children. Furthermore, even when the closer parental consanguinity (first-degree cousins) was considered, statistically significant risk was not found. In addition, when the data were analyzed in a different way, parents of asthmatic children did not have significantly higher consanguinity rate than the parents among controls. This observation was true for both overall and first-degree cousin consanguinity.

The literature on the interaction between consanguinity and asthma is scarce. In a study from the United Arab Emirates (a country with a high rate of consanguinity), Abdulrazzaq *et al.* reported that although the occurrence of asthma was more common in the offspring of consanguineous marriages (7.9% vs. 6.9%), the difference was not statistically significant.[10] In another study from Kuwait, the consanguinity rate in the parents of children with asthma was 42%, which is lower than the 56% rate reported in the general population.[11] The findings of both of these studies are in agreement with ours suggesting that there is no increased risk of asthma in children of consanguineous marriages. However, a study from Qatar (another neighboring country with a high rate of consanguinity), reported a significantly higher proportion of asthma in the offspring of consanguineous couples ((23.1 vs. 12.1) with a relative risk of 1.37, confidence interval: 1.24 – 1.51 and *P*-value of <0.001.[12] The reasons for the discrepancy in the results of these studies are not clear. A possible explanation may be related to sample size, which is smaller in the studies from the Emirates, Kuwait and ours. Another possible explanation may be related to the epidemiological variation of bronchial asthma with age suggesting an increasing role of genetic factors with increasing age;[13] a difference in the age distribution of study populations is a likely explanation. Genetic and environmental exposure variations are unlikely as the populations of these studies originate from the same area and live in a close geographical location. In conclusion, our findings suggest that parental consanguinity does not increase the risk of bronchial asthma in Saudi Arab children. Further studies on the role of consanguinity in the occurrence of specific conditions, including bronchial asthma, involving a larger number of cases and controls are needed to provide more focused counseling.
